# Engineered yeast *Yarrowia lipolytica* as a chassis for biosynthesis of fatty acids from mannitol and macroalgal biomass extracts

**DOI:** 10.1186/s12934-025-02699-9

**Published:** 2025-03-26

**Authors:** Mateusz Szczepańczyk, Dorota A. Rzechonek, Adam Dobrowolski, Aleksandra M. Mirończuk

**Affiliations:** 1 Wrocław University of Environmental and Life Sciences, Institute of Environmental Biology, Laboratory for Biosustainability, 5b Kozuchowska St, Wroclaw, 51-631 Poland; 2 Department of Life Sciences (LIFE), Chalmers University of Technology, Kemivägen 10, Göteborg, SE-412 96 Sweden

**Keywords:** Mannitol utilization, Yeast, Macroalgae biomass, Fatty acid synthesis

## Abstract

**Background:**

*Yarrowia lipolytica* possesses the capability to utilize many unconventional carbon sources, such as crude glycerol, alkanes and fatty acids. Despite producing polyols, such as erythritol, arabitol and mannitol, the re-utilization of mannitol is not as efficient as erythritol utilization. Genes involved in mannitol uptake and metabolism in *Y. lipolytica* remain undescribed. However, deletion of the *EYD1* gene (*YALI0F01650g*), believed to encode erythritol dehydrogenase, has been found to result in a high rate of growth on media containing mannitol as the sole carbon source. Therefore this unique feature was used for further fermentation studies on media containing macroalgal mannitol extracts, obtained from the brown alga *Fucus vesiculosus*, to produce value-added products.

**Results:**

The obtained strain *AJD Δeyd1Dga1* was able to uptake pure and algal mannitol efficiently and produce high amounts of lipids, thanks to overexpression of the *DGA1* gene (*YALI0E32769g*), encoding diacylglycerol (DAG) acyltransferase. The lipid content reached almost 32% of the overall dry biomass as compared to the wild type strain, where this value was more than 4 times lower. Additionally, the biomass at the end of the experiment was the highest among all of the tested strains, reaching 12.67 g/L, more than 50% higher than the control strain.

**Conclusions:**

The results of this study shed new light on the potential for the yeast *Y. lipolytica* to utilize macroalgae biomass as a carbon source for production of value-added products, including biomass and lipids. Moreover, the increased mannitol utilization capabilities can provide new insight into mannitol metabolism, including its uptake, which is especially crucial, as the metabolic pathways for all polyols produced by this organism seem to be closely intertwined.

**Supplementary Information:**

The online version contains supplementary material available at 10.1186/s12934-025-02699-9.

## Background

The increase of the human population and the constant demand for energy are a driving force behind the development of new types of energy sources, which could be an alternative for the depleting reserves of fossil fuels. One of the alternatives is the usage of biomass, a potential feedstock for generation of biofuels [1]. Algal biomass is considered to be a third-generation form of biomass [8, 49]. Algae present many advantages over both first- and second-generation biomass. They possess the ability to capture high quantities of carbon dioxide, do not compete for agricultural land and are characterized by one of the highest growth rates among photosynthetic organisms [24, 49]. In addition, they have an ability to synthesize oil for biofuel production. However, the algal oil is highly unsaturated, which makes it more volatile in high temperatures [51]. Algal carbohydrates, which constitute a high percentage of the overall dry weight, can be easily processed to bioethanol [24], due to the absence of lignin, which makes the process of saccharification require pretreatment. This type of biomass has disadvantages as well, such as very diverse chemical composition among the species. The wide range of present carbohydrates and their composition among species complicates efforts to compile a universal protocol for extraction and then fermentation of the extracts [6].

Brown algae, belonging to the third generation of biomass, are a large and diverse group of multicellular organisms with the number of species estimated to be over 1.8 thousand. Almost all of them are exclusively marine and inhabit the intertidal zone [18, 48] and do not produce lignin [24], in contrast to land plants. Brown macroalgae are characterized by a complex carbohydrate composition, consisting of alginate, fucoidan, laminarin and mannitol, the latter two acting as carbohydrate reserves, for storage during the summer months [6]. The concentration of storage mannitol reaches up to 30% of the dry biomass, depending on the species [36]. Additionally, mannitol may act as an osmoprotectant or an osmolyte, as suggested by research on *Ectocarpus siliculosus* [11]. Based on a World Bank report, production of macroalgae is steadily increasing, from 10.6 million tons in 2000, to 20.1 million tons in 2010, up to 35 million tons in 2020. The global market for macroalgae is expected to increase by a rate of 2.3% annually, to reach 11.8 billion dollars in 2030. Most of the produced algae are used for human consumption, animal feed in aquacultures or additives for pet food. However, alternative applications of macroalgae have been proposed, such as production of bioplastics and other commercially advantageous compounds, mostly due to the algae complex composition [50].

The amount of mannitol in dry biomass for brown algae species was characterized by Reed et al. [36]. In *F. serratus* the percentage of mannitol reached 7.1% of dry biomass and 5.5% for *F. vesiculosus* [36]. The concentration of storage polyol is a subject of seasonal fluctuations and is based on the salinity of the environment. For instance, mannitol content for *F. vesiculosus* was recorded as 8.1% of dry biomass in a recent study [31]. Different parts of the algae also contain varying amounts of mannitol [21, 25]. A significant advantage of the usage of brown algae as a mannitol source is not only the high concentration of the polyol but also an easy extraction method. Mannitol can be extracted from the algal biomass without the need of enzymes, by solid-liquid extraction with or without diluted acid solution [6]. Additionally, thermal treatment is used to obtain the algal extract for fermentation studies [26].

*Yarrowia lipolytica* is an oleaginous yeast that can withstand unfavorable conditions, such as low pH and high salinity [37, 41]. In addition, those conditions promote the synthesis of polyols, which acts as osmoprotectants [45] Y. *lipolytica* has an ability to utilize many unconventional carbon sources [40] and produce a wide range of value-added products, which makes this organism an object of interest in multiple research areas [9, 14, 35, 37]. One of the carbon sources less commonly utilized by microorganisms is mannitol. *Y. lipolytica* possesses the ability to take up mannitol from the medium, but the process is not efficient [44]. The molecular pathway of mannitol utilization is still under debate, but some genes involved in this process have been described [43, 52]. More focus is put on the polyol synthesis pathway, and therefore detailed information about mannitol catabolism is not available [47]. It is necessary to create more efficient strategies of mannitol utilization by various microorganisms, to fully achieve its potential. Mannitol is one of the most common carbohydrates in the marine algae, yet the knowledge about the metabolism and uptake of this polyol is limited. Effective strategies of genetic modifications of microorganisms can lead to the improvement of mannitol utilization and conversion, since species such as *S. cerevisiae* have been shown to grow on mannitol only after long adaptation on media with this polyol [23].

The pathway for fatty acid synthesis in *Y. lipolytica* is already well studied, as this yeast is a model organism for lipid synthesis in eukaryotic cells [33, 34]. The *DGA1* gene (*YALI0E32769g*), which encodes acyl-CoA diacylglycerol acyltransferase, and its overexpression were proved to have a significant impact on lipid synthesis and accumulation within the cells [3, 46]. The tolerance of high osmotic pressure and a wide pH range, coupled with the GRAS status and ability to produce a diverse array of value-added products from waste materials, makes *Y. lipolytica* a promising host for fermentation studies on algal mannitol extracts [13, 27]. The possibility to modify the fatty acid synthesis pathway leading to the accumulation of lipids in the cells further encourages development of a method of conversion of algal mannitol to microbial oils with the usage of *Y. lipolytica* [12].

In this study the engineered yeast *Y. lipolytica* was used for efficient mannitol utilization, leading to high production of biomass. The aim of the further modification was to produce large quantities of fatty acids, with mannitol as a carbon source. As the utilization of pure mannitol for production of other value-added products is economically dubious, here we used a medium containing mannitol flushed out from commercially available macroalgae *F. vesiculosus* biomass. The obtained data suggest that the engineered yeast is a suitable host for fatty acid synthesis from macroalgal biomass.

## Materials and methods

### Strains, media and culture conditions

The bacterial and yeast strains used in this study are listed in Table 1.

**Table 1:** Bacterial (*E. coli*) and yeast (*Y. lipolytica*) strains used in this study

Strain	Genotype or Plasmid	Source
***E. coli***		
DH5α	F − endA1 glnV44 thi-1 recA1 relA1 gyrA96 deoR nupG Φ80dlacZΔM15 Δ(lacZYA-argF)U169, hsdR17(rK-mK+), λ–	[19]
DH5α	pUC-URA	[39]
DH5α	pUC-URA-EYD1g	[41]
DH5α	pAD_UT-DGA1g	[12]
***Y. lipolytica***		
AJD	*MATA*, A101: ura3-302	[29]
AJD ΔEYD1	*MATA*, A101: ura3-302 *ΔYALI0F01650g*	[41]
AJD ΔEYD1 ura-	*MATA*, A101 ΔEYD1	[41]
AJD ΔEYD DGA1	*MATA*, A101: ura3-302 ΔEYD, overexpression of *YALI0E32769g*	This study
AJD DGA1	*MATA*, A101: ura3-302, overexpression of *YALI0E32769g*	[12]

*E. coli* strains were grown in LB (Luria Bertani) medium (A&A Biotechnology, Gdańsk, Poland) at 37 °C. Addition of ampicillin (100 mg/L) was required to screen for transformants on solid LB plates (2% agar).

*Y. lipolytica* strains were grown in YPD medium (A&A Biotechnology) at 28 °C. The inocula for plate reader analysis and lipid synthesis experiments were grown in YPD medium for 24 and 48 h respectively. Lipid synthesis was carried out in lipid synthesis medium, which is a modified yeast nitrogen base (YNB) medium, containing 5% mannitol, 0.5% YE and ammonium sulfate, to obtain the C/N ratio of 60. Similarly, the medium containing mannitol extracted from algae was supplemented with ammonium sulfate to obtain the C/N ratio of 60 and 0.5% YE. Initial analysis was conducted in media containing 2% and mannitol, with the amount of ammonium sulfate adjusted to that value. Mannitol utilization was carried out in YNB medium with 5% mannitol.

The deep-well plate experiments were carried out in 24-well plates with 3.5 mL of the working volume. The plates were incubated at 28 °C with constant orbital shaking (200 rpm). The shake flasks experiments were conducted in the same conditions.

### Mannitol extraction

One hundred grams of dried *Fucus vesiculosus* (Dary Podlasia, Poland) was placed in a 2 L beaker and soaked in 500 ml of water. The content of the beaker was under constant agitation via a mechanical stirrer (CAT R50, Germany). The samples were collected for the HPLC analysis, to determine the time required for the maximum possible extraction in those conditions. After two hours, the remaining extract was transferred to 50 ml tubes and centrifuged for 15 min at 5000 rpm to remove particles of algae, which could affect the growth of the yeast and change the parameters of the medium. The extract was then used to soak the algae again for two hours under constant agitation, maintaining the 1:5 ratio of algae to liquid. The obtained rinse was again centrifuged and a sample for HPLC analysis of mannitol content was taken. After the centrifugation, the supernatant was transferred to glass bottles and sterilized (121 °C, 15 min). Mannitol extracts were additionally centrifuged at 15 000 rpm for 10 min at room temperature.

### Growth analysis

The strains were grown in YPD medium for 24 h in 100 ml flasks, filled with 10 ml of the medium. One milliliter of the culture was then centrifuged in a 1.5 ml Eppendorf tube for 3 min at 5000 rpm. The medium was discarded and the cells were resuspended in 1 ml of sterile water. Growth analysis was conducted in 96-well plates in 200 µl of the medium. The initial OD was set at 0.1. The culture parameters were set at 28 °C, constant orbital agitation (200 rpm) for 30 min with the stop 5 s before the measurement took place. Growth of the strains was monitored by measuring the optical density at the range of 600 nm for a total of 72 h. All of the analyses, including un-inoculated media, were performed in triplicate. The analysis was conducted in TECAN Infinite M Nano (Switzerland).

### Genetic modification

Overexpression of the *DGA1* gene (*YALI0E32769g*) was carried out after the marker Ura3 gene was successfully eliminated via the cre-lox method from the AJD *Δeyd1* strain [41]. The procedure followed the proposed protocol as described by Fickers et al. (2003). The transformation of the AJD *Δeyd1* ura- strain was carried out by the lithium acetate method. The overexpressing plasmid was created by Dobrowolski et al. [12].

### HPLC analysis

The samples obtained from the deep-well plates and shake flask experiments were centrifuged for 15 min at 15,000 rpm. Supernatants were carefully transferred to new Eppendorf tubes, not disturbing the pellet, and the clear supernatant was additionally centrifuged again for 10 min at 15 000 rpm. To characterize the concentration of mannitol and other polyols (erythritol and arabitol) and citric acid, the centrifuged samples were diluted 10 times and analyzed by HPLC analysis using Ultimate 3000 HPLC with a HyperRez Carbohydrate H + column (Thermo Scientific), coupled to a UV detector (λ = 210 nm) (Dionex, USA) and a refractive index detector (Shodex, Japan). For the elution 25 mM trifluoroacetic acid at 65 °C with a flow rate of 600 µl/min was used.

### Biomass analysis

The post-culture liquid was centrifuged (10 min, 5000 rpm), and the cellular pellet was washed with distilled water and run through the filtration system. Nitrocellulose membrane (0.22 μm, GSWP) allowed for the filtration of biomass. The obtained biomass was dried on a nitrocellulose disc for 24 h and then weighed in a drying scale (RADWAG model MA 50/1.R.WH, Poland) at 105 °C. To analyze the biomass content from the shake-flask experiments, 10 ml of the culture was run through the filtration system, while 1 ml of the medium was used in the case of the deep-well plates.

### Lipid content analysis

Lipid synthesis was conducted in lipid synthesis medium containing: YNB (1.7 g/L), yeast extract (5 g/L), mannitol and ammonium sulfate in a C: N ratio of 60. The media were prepared with pure mannitol and algal mannitol extracts; therefore the amounts of both mannitol and ammonium sulfate were adjusted to the values of mannitol obtained in the mannitol extracts from various algal species shortly before sterilization. The lipid production analysis was conducted in lipid synthesis medium in 300 ml Erlenmeyer flasks with 50 ml of the working volume. The OD_600_ of the culture was set at 1.0. Analysis of the lipid content of the biomass grown in medium with algal mannitol was carried out in deep-well plates in 3.5 ml of the working volume.

The biomass from the fermentation study was lyophilized for fatty acid production analysis. Ten to twenty milligrams of biomass was mixed with 2 ml of 2.5% sulfuric acid in methanol, with an internal standard of C17:0 (50 mg/ml). The extraction was carried out in glass tubes with Teflon caps. After mixing for 2 min, the samples were incubated for 90 min at 80 °C. Extraction of fatty acid methyl esters was conducted after addition of 1 ml of hexane and 0.5 ml of water. The organic phase with fatty acids appeared after mixing and centrifugation. This phase was then transferred into glass vials for GC analysis on GC-2010 Plus apparatus (Shimadzu, Japan) with a flame ionization detector (FID) and autoinjector (AOC-20i). The separation was performed on a 70% cyanopropyl polysilphenylene siloxane column TR-FAME, 30 m × 0.32 m × 0.25 μm. The temperature for the injector was set at 270 °C and 280 °C for the detector. Helium was used as the carrier gas (flow of 1.52 ml/min). The identification of the fatty acids was possible due to the reference standard (Supelco 37 Component Fame Mix).

## Results

### Growth analysis of *Y. lipolytica* strains on mannitol as a sole carbon source

*Y. lipolytica* possesses the ability to produce polyols as a response to conditions evoking osmotic stress [28]. These polyols can also be utilized as a carbon source, which is an issue in the production of these compounds on an industrial scale. Analysis of the erythritol metabolism pathway in *Y. lipolytica* led to characterization of many genes involved in this process [4, 22, 39] including the gene *EYD1* (*YALI0F01650g*). The strain with knockout of this gene was unable to grow on erythritol as a sole carbon source [4]. However, the metabolic pathway of mannitol in this yeast remains unclear. Interestingly, production of mannitol can be increased by optimised fermentation conditions or by metabolic engineering by deletion of a set of erythrose reductases involved in erythritol synthesis [45].

**Fig. 1:**
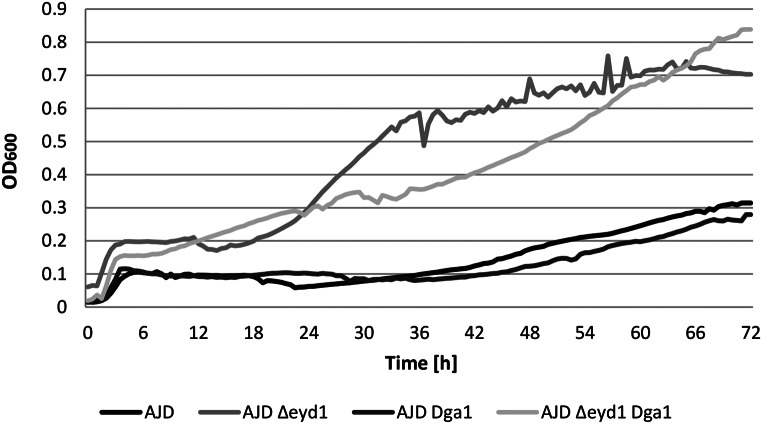
Growth curves for the obtained strains and the parental strain (AJD) on minimal medium containing 2% mannitol (YNB + 2% mannitol). The analysis was performed in triplicates

Since deletion of the *EYD1* gene leads to inability to use erythritol as a sole carbon source, the question of whether the same will be true with other polyols was addressed. To this aim, the strain with the *EYD1* gene knocked out was tested on media containing different carbon sources. The goal was to determine the influence of the knockout on the uptake of atypical media components, including mannitol and arabitol (data not shown). The wild type strain acted as a control and since the engineered strain was foreseen as a host for lipid production, overexpression of the *DGA1* gene was introduced, resulting in the strain AJD *Δeyd1 Dga1*. The results of yeast strains’ growth are shown in Fig. 1.

Interestingly, the analysis proved that the knockout of *YALI0F01650g* not only leads to the inability to utilize erythritol [4] but also improves the growth on mannitol. Both strains with *EYD1* gene knockout grew well on mannitol as a sole carbon source, while the WT strain and AJD *dga1* could hardly grow under these conditions. The lag phase was extended to 36 h for both of these two strains. Additional changes in the medium regarding the concentration of mannitol revealed that the trend observed for the YNB medium with 2% mannitol was maintained in conditions with mannitol concentration up to 10%. This result indicates that there is no need to limit the concentration of mannitol in the media to support the growth of the strains. Moreover, the growth in rich medium (YPD) did not indicate any differences between the strains (Supplementary Data, Fig. 1); therefore the potential impact of genetic modifications on yeast growth was initially ruled out.

**Fig. 2:**
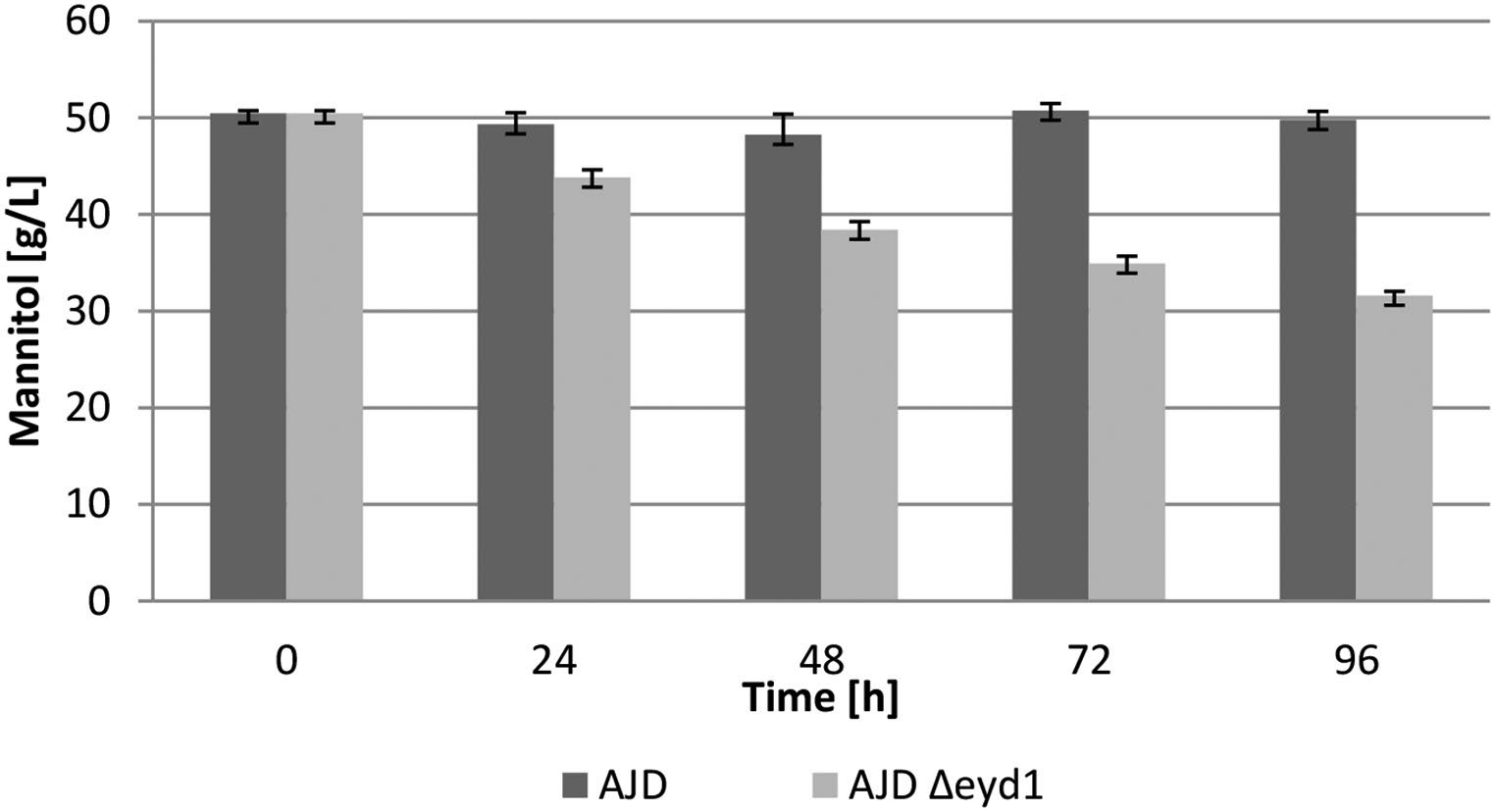
Mannitol utilization in YNB medium with 5% mannitol over 96 h period. WT strain– black, AJD *Δeyd1* strain– green

The next step was the analysis of mannitol utilization in YNB medium in flasks, to test the utilization parameters and to increase the scale of the process. The strains were grown in YPD (yeast extract, peptone and dextrose) medium for 24 h and then in YNB medium with 5% mannitol. Mannitol utilization was measured every 24 h for 96 h as shown in Fig. 2. The results indicate that the WT strain is unable to utilize mannitol in those conditions, while the knockout strain utilizes 19 g/L of mannitol within 96 h of the experiment. This allows utilization of the modified strain in the conversion of mannitol and shows its potential in synthesis of value-added products.

### Lipid accumulation in medium with pure mannitol

As the AJD *Δeyd1* and AJD *Δeyd1 Dga1*strains were proven to be able to take up mannitol, the ability to produce value-added products from this carbon source was analyzed. Lipids are produced in large quantities by *Y. lipolytica* under nitrogen limitation conditions with an excess of carbon; therefore lipid production by yeast in YNB medium supplemented with 2% pure mannitol was analyzed. First, the strain cultures were grown over-night in YPD medium and subsequently inoculated in medium containing 20 g/L of mannitol (YNB + 2% mannitol). Ammonium sulfate was added to obtain the ratio C/N 60. Additionally 0.5% yeast extract was supplemented to improve the growth of strains unable to utilize mannitol in the minimal medium as well as to support the growth at the beginning of the process.

**Fig. 3:**
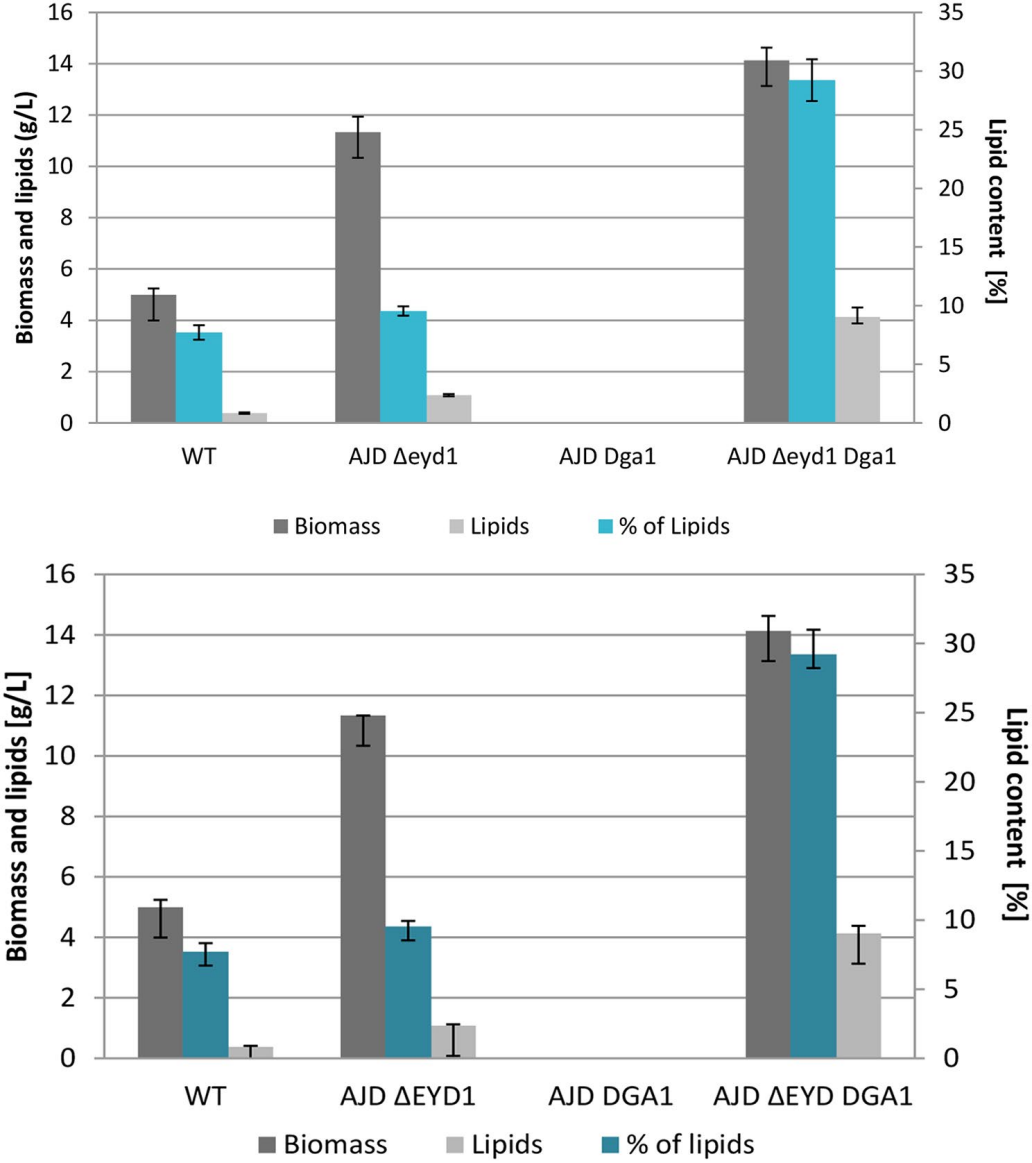
Biomass (gray) lipid production (light gray) and percentage of produced lipids (teal) in strains grown on mannitol as a sole carbon source after 72 h of cultivation, performed in triplicate. WT strain, strain with *EYD1* knockout, knockout of *EYD1* gene with overexpression of *DGA1* gene grew on the medium with pure mannitol, while AJD *Dga1*did not grow efficiently enough to analyze the biomass and lipid content level

The obtained results proved that strains with the *EYD1* gene deleted grew significantly better than the control strain. Strain AJD *Δeyd1* achieved 11.33 g/L and AJD *Δeyd1 Dga1* 14.13 g/L of biomass, the control strain only 4.99 g/L (Fig. 3), which could be possible by the supplementation of the medium with yeast extract, as the strain was unable to utilize mannitol in the previous experiment as depicted in Fig. 2. The biomass could not be determined for the AJD *Dga1* strain, as it failed to grow efficiently enough in this type of medium, and therefore the analysis of lipid production was also impossible to perform. Moreover, due to higher biomass production, which is a result of the improved carbon uptake, strains with *EYD1* knockout additionally accumulated more lipids at the end of the cultivation. That was caused by the shift of the carbon flux towards lipids and connected with the carbon to nitrogen ratio. Lipid production in the strains tested on pure mannitol revealed that the strain with knockout of the *EYD1* gene produced only slightly more lipids than the control strain, despite the ability to utilize mannitol more efficiently than the wild type strain (Fig. 3). Additional overexpression of the *DGA1* gene improved the lipid accumulation to up to 29.22% of dry biomass, which corresponds to 4.13 g/L of lipids, while the WT strain reached 7.71%, yielding 0.38 g/L of lipids for this strain (Figure S1). The yield of lipid production was calculated based on utilized mannitol. It was characterized as 0.038 g/g for the WT strain, increased to 0.109 g/g for the AJD *Δeyd1* strain and reached a value three times higher (0.332 g/g) for the AJD *Δeyd1 Dga1*strain.

### Lipid production in media with algal mannitol

**Fig. 4:**
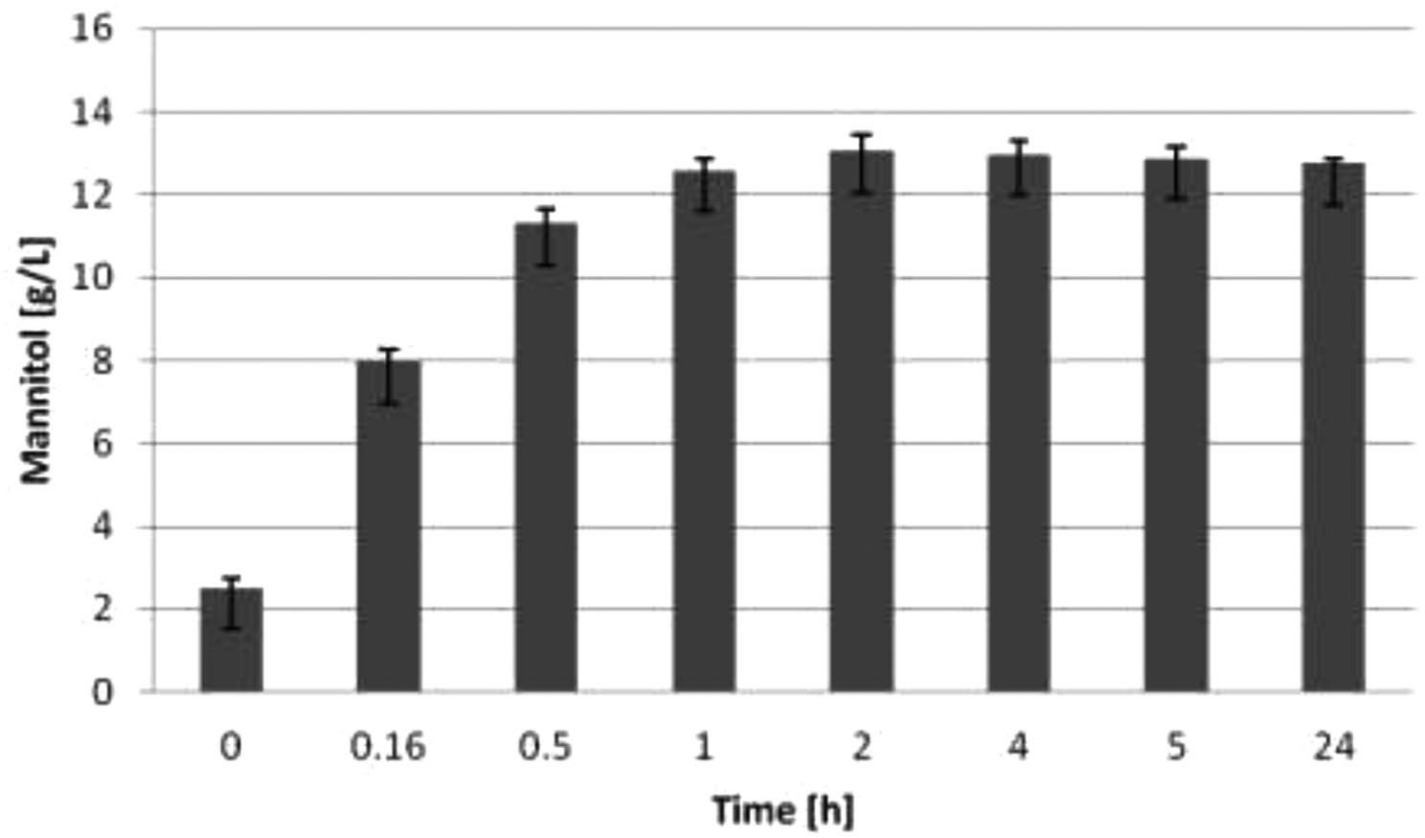
Concentration of mannitol in algal rinse over time

Since the utilization of pure mannitol proved to be effective for the *EYD1* knockout strains, the following experiments were conducted on mannitol from brown macroalgae. With the procedure described in this study, it was possible to obtain the mannitol rinse from *F. vesiculosus* within 1–2 h. Samples were collected to determine the mannitol concentration over time, to stop the extraction process immediately after the concentration of mannitol stopped increasing (Fig. 4). The results indicate that running the extraction process over two hours yielded no more mannitol; therefore the extraction was stopped after this time point.

**Fig. 5:**
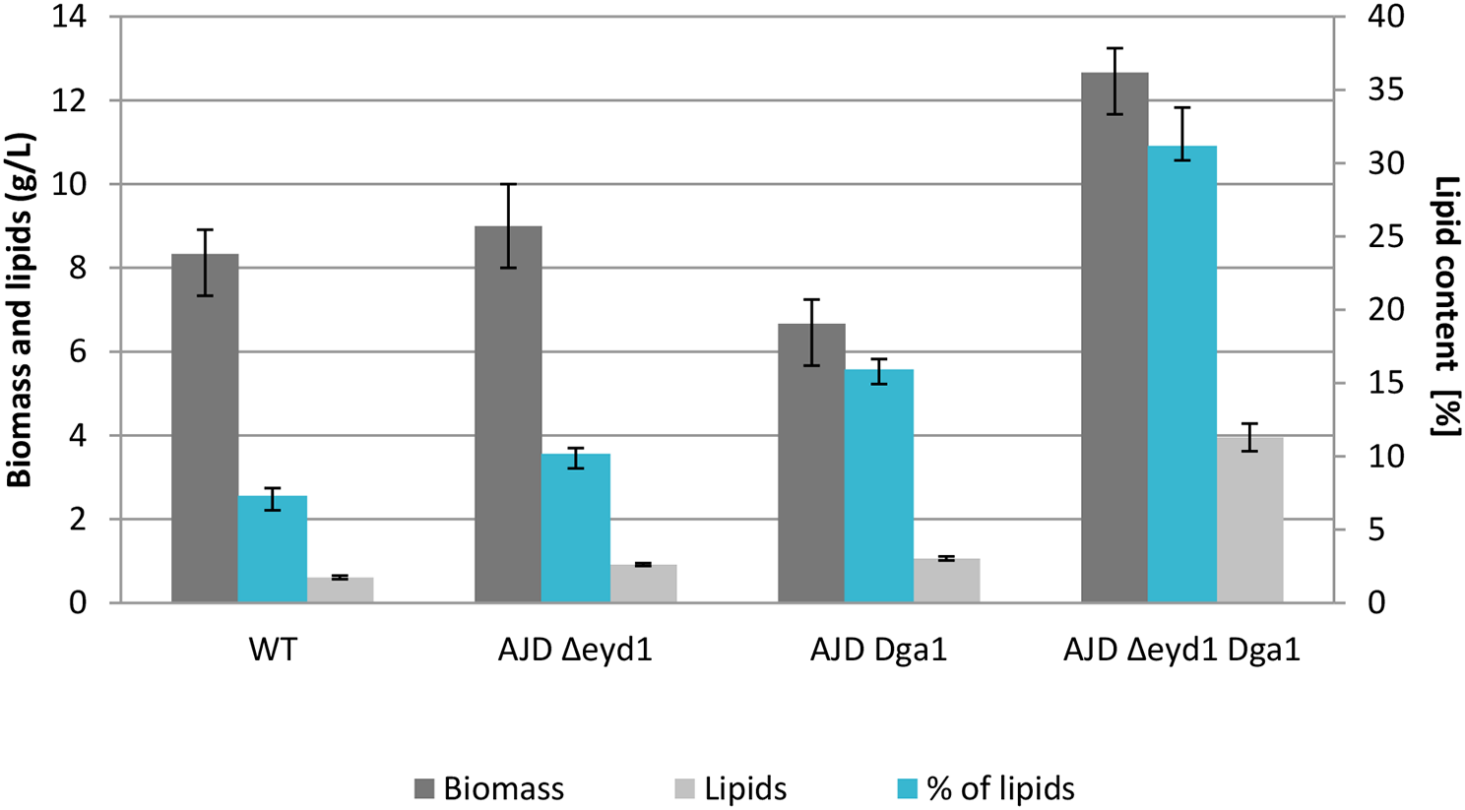
Biomass (gray), lipids (light gray) and percentage of produced lipids (teal) in the medium with algal mannitol extracts in deep-well plates, data gathered from triplicates after 72 h of cultivation

Next, the growth experiment was done in deep-well plates on medium from brown macroalgae (Fig. 5). Mannitol extraction was performed on *F. vesiculosus*, which was found to accumulate up to 8.1% mannitol in dry biomass [31]. This species was selected due to its availability, low price and minimal share in the food market as compared to other species grown on a large scale in algal farms. However, different brown algae species, with higher concentration of mannitol in the dry biomass could be used to obtain rinse more rich in mannitol. The macroalgal rinse was used as a base for the medium with supplementation of YNB. Based on the previous experiments, ammonium sulfate and yeast extract were added to the medium. Biomass and lipid content analysis revealed that the AJD *Δeyd1 Dga1*strain reached the highest biomass and lipid production level, as shown in Fig. 5. The lipid content for this strain was measured at 3.95 g/L, exceeding the value for the WT strain by 3.3 g/L. The biomass reached 12.67 g/L. The AJD *Dga1* strain had the lowest biomass after the fermentation study (6.67 g/L) but the second-highest lipid content (1.06 g/L). The strain previously failed to grow on a medium with pure mannitol as a carbon source. However, most likely, the macroalgae-based medium contains organic matter that can be used by yeast as a carbon source. *Y. lipolytica* strain *Δeyd1* obtained slightly higher biomass and lipid production than the WT strain, 8.33 g/L and 9 g/L, respectively. The yield of lipid production was calculated taking into consideration the utilized algal mannitol throughout the experiment. The lowest yield was for the AJD *Δeyd1* strain (0.047 g/g), which also utilized the highest amount of mannitol from the medium. The WT strain utilized only 3.84 g/L of mannitol, so the yield of lipid production was higher and reached 0.159 g/g, as the lipids could be produced as a result of utilization of other carbon sources in the medium, mainly the components of yeast extract and residual citric acid [38].

Similarly to the production in media based on pure mannitol, the strain AJD *Δeyd1 Dga1*achieved the highest percentage of lipids in dry biomass (31.19%), as the strain possesses the ability to utilize mannitol most efficiently and convert it into storage lipids. Significantly, the AJD *Dga1* strain achieved the second highest percentage of lipids (15.93%), while it failed to grow on medium with pure mannitol (Figure S1). The wild type strain accumulated similar amounts of lipids in dry biomass in macroalgae-based media and YNB supplemented with mannitol. The same was observed for the AJD *Δeyd1* strain. Similarly, the AJD *Dga1* strain utilized 2.44 g/L of mannitol during the course of the fermentation, which led to productivity of 0.435 g/g. The yield for the AJD *Δeyd1 Dga1*strain reached 0.237 g/g.

Finally, we tested the fatty acid profile of the engineered strains growing on two different carbon sources: pure mannitol and algal mannitol (Table 2). The most abundant fatty acid was oleic acid (18:1). The content of oleic acid ranged from 48.4% for the WT strain in medium with algal mannitol, to 73.3% for the AJD *Δeyd1 Dga1*strain on the same medium. Accumulation of this fatty acid was lower for the WT strain and AJD *Δeyd1* strain on algal mannitol medium than for pure mannitol minimal medium, dropping by 7 and 8% respectively. The WT strain produced 21.9% of the overall fatty acids in the form of palmitic acid (16:0) in algal mannitol medium, exceeding the value by over 10% as compared to the pure mannitol medium. Interestingly, this strain produced stearic acid (18:0) on pure mannitol (7.6% of the overall production), but none was detected in the algal extract medium. In other strains this acid was observed (4.5-8%) regardless of the medium. None of the fatty acids produced by the AJD *Δeyd1 Dga1*strain, except for oleic acid, crossed the 10% level in both analyzed media.

**Table 2:** Percentage of lipids produced by tested strains in medium with pure mannitol and algal mannitol extracts. C16:0– palmitic acid, C16:1– palmitoleic acid, C18:0– stearic acid, C18:1– oleic acid, C18:2– linoleic acid, nd– not detected

	Pure mannitol			Algal mannitol extracts		
	WT	AJD *Δeyd1*	AJD *Δeyd1 Dga1*	WT	AJD *Δeyd1*	AJD *Δeyd1 Dga1*
C16:0	11.91 (± 0.24)	8.88 (± 0.85)	5.77 (± 0.52)	21.96 (± 0.99)	12.13 (± 1.02)	6.90 (± 0.33)
C16:1	8.59 (± 0.58)	7.13 (± 0.42)	7.16 (± 0.94)	11.67 (± 0.86)	10.77 (± 0.48)	8.36 (± 0.13)
C18:0	7.56 (± 0.29)	5.52 (± 0.71)	8.02 (± 0.73)	nd	7.04 (± 0.69)	4.55 (± 0.26)
C18:1	55.08 (± 1.29)	68.59 (± 1.4)	72.52 (± 2.53)	48.39 (± 2.57)	56.66 (± 2.35)	73.29(± 0.42)
C18:2	16.86 (± 0.86)	9.88 (± 0.47)	6.54 (± 0.41)	17.99 (± 0.94)	13.41 (± 0.16)	6.91 (± 0.21)

### Scaling up of lipid production with algal mannitol medium

**Fig. 6:**
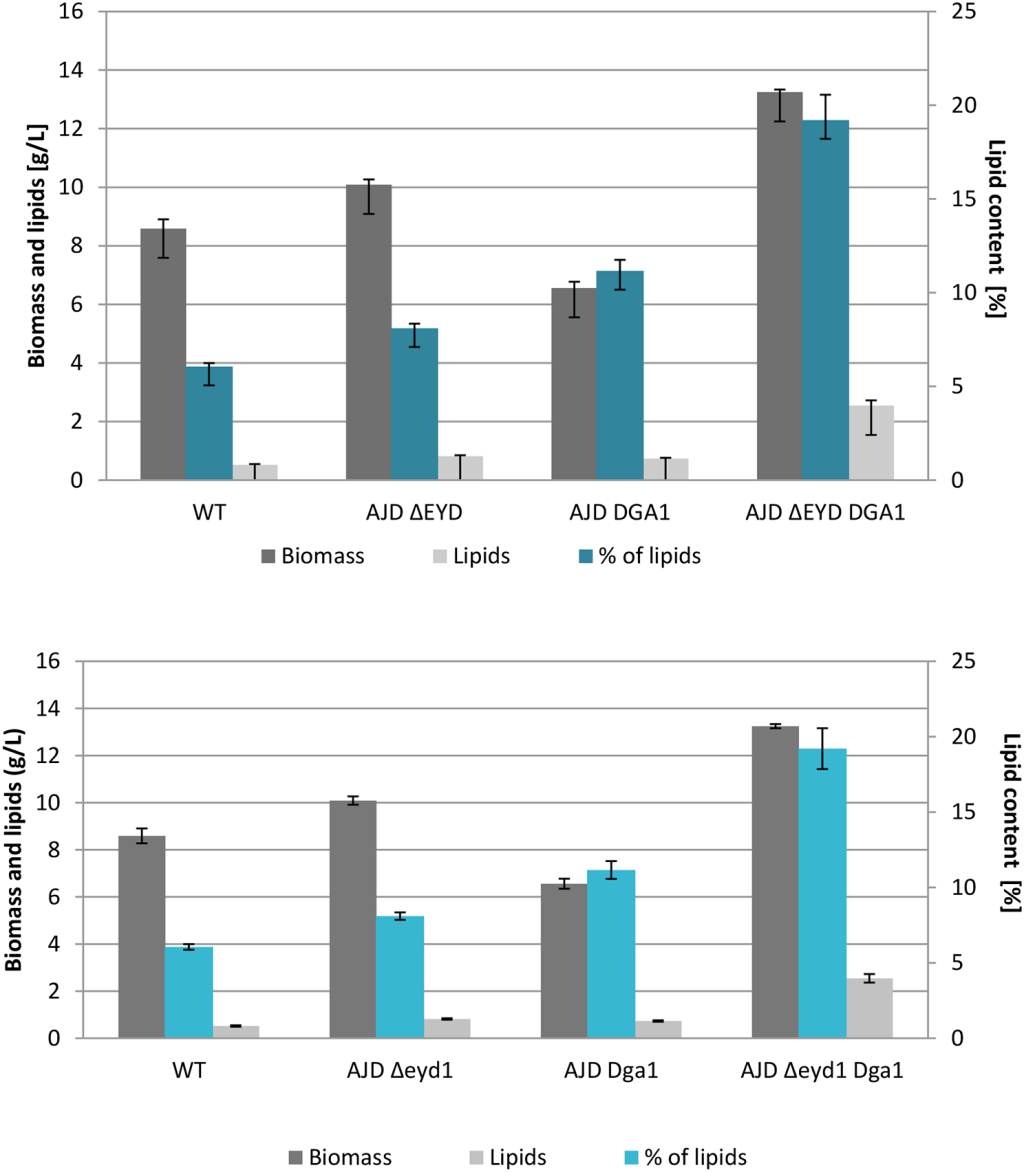
Biomass (gray) lipid production (light gray) and percentage of produced lipids (teal) in strains grown on mannitol as a sole carbon source

The process was then scaled up to 30 ml of working volume in 300 ml flasks with medium supplemented with yeast extract, YNB and ammonium sulfate. Mannitol was nearly depleted within 72 h of the culture for AJD *Δeyd1 Dga1*, at which point the process was stopped to avoid breakdown of the storage lipids. Biomass was also analyzed and it reached up to 13.2 g/L for the strain with overexpression of *DGA1* and knockout of *EYD1*, while the biomass for strain with the knockout reached 10 g/L. The WT strain produced 8.59 g/L, while the strain AJD *Dga1* had only 5.56 g/L, in agreement with the previous experiment. The percentage of lipids in dry biomass was the highest for AJD *Δeyd1 Dga1* at 19.21%, AJD *Δeyd1* obtained 8.10%, AJD *Dga1* 11.16%, and the control strain only 6.06% (Fig. 6). Therefore the AJD ΔEYD DGA1 strain accumulated more than three times the amount of lipids of the WT strain and more than double that of AJD *Δeyd1*, while simultaneously obtaining the highest biomass among the tested strains. The highest yield was observed for the AJD *Dga1* strain (0.232 g/g), while the yield for the AJD *Δeyd1 Dga1* strain was 0.151 g/g. AJD *Δeyd1* and WT obtained 0.043 g/g and 0.091 g/g, respectively.

**Fig. 7:**
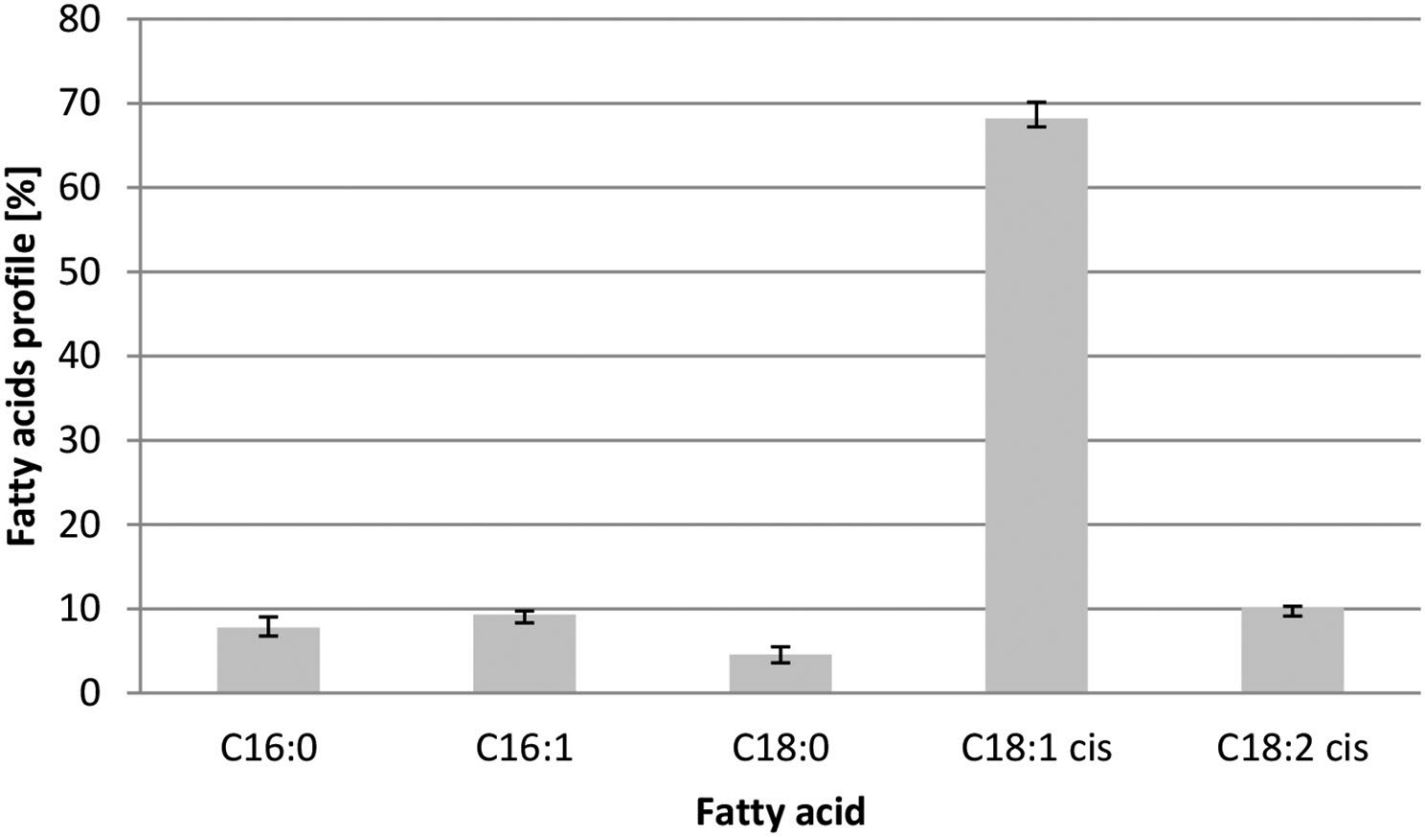
Fatty acid profile for AJD *Δeyd1 Dga1*strain in algal mannitol medium in shake flasks. C16:0– palmitic acid, C16:1– palmitoleic acid, C18:0– stearic acid, C18:1– oleic acid, C18:2– linoleic acid

The profile of the fatty acids produced by the strain AJD *Δeyd1 Dga1*was also investigated and depicted in Fig. 7. The most prevalent was oleic acid, which represented nearly 70% of the overall produced lipids, while stearic acid was produced in the lowest amount, reaching 4.57%. These results are similar to those obtained on a smaller scale, revealing a trend, where the overall production of oleic acid reached values over 70%, while stearic acid constituted 4.55%. Therefore scaling up of the process does not affect the fatty acid profile significantly.

## Discussion

Despite multiple years of research on polyol metabolism in *Y. lipolytica*, most of the knowledge concerns erythritol, while mannitol and arabitol remain relatively unexplored. There have been multiple attempts to decode the complete pathway of mannitol synthesis and transport. The most prominent research on this topic is the discovery of mannitol dehydrogenase [47], disruption of which leads to impairment of mannitol production from fructose, while the parameters remain the same for more commonly used carbon sources: glycerol and glucose. Two other potential mannitol dehydrogenases encoded by *YALI0D18964g* and *YALI0E12463g* were also proposed based on their similarity to the previously described *YALI0B16192g* [16]. The research on *Saccharomyces cerevisiae* revealed the potential transporters of mannitol as Hxt13 and Hxt17, but their homologs in *Y. lipolytica* genome are yet to be confirmed [23]. Production of mannitol can be increased by other means, including increased fermentation temperature, with overexpression of heat shock proteins, adjusting the osmolarity of the media and the pH, but the true mechanism behind the impact of these processes on mannitol metabolism is still largely unknown [52]. However, the analysis of overcoming the stress conditions by *Y. lipolytica* suggested that mannitol could serve as the uniform stress response molecule, due to its wide variety of functions [5].

*Y. lipolytica* possesses the ability to utilize many unconventional carbon sources, yet the utilization of mannitol is not efficient. Studies have indicated that this polyol is utilized after being produced by the yeast as a response to osmotic stress, when other carbon sources are depleting [44]. Moreover, the metabolic pathways of arabitol, mannitol and erythritol seem to be closely linked. It is prominent when the erythritol synthesis pathway is impaired, leading to the increase in production of arabitol and mannitol [45]. Additionally, transcription factor related to erythritol utilization (EUF1) affects many processes in the yeast *Y. lipolytica*, including genes encoding potential polyols transporters [40]. This could result in a shift of potential to utilize different polyols. However, the usage of mannitol as a sole carbon source is not an efficient strategy for production of biomass or value-added products. Polyols are not used as a carbon source in industrial scale fermentations using *Y. lipolytica*, due to their high price and possible application in the food and pharmaceutical industries. The possibility to utilize mannitol from algal extracts to produce value-added products is a potential strategy to use the algal biomass efficiently. To date, studies have focused mostly on production of bioethanol from algal extracts by microorganisms, including thermophilic *Clostridia* [6]. Other microorganisms, such as *Zymobacter palmae*, have been utilized to produce ethanol from mannitol in an oxygen-limited conditions, yielding 0.38 g of ethanol per 1 g of mannitol [20]. More studies were also performed with microalgae, which can be grown in a co-culture with yeast [2]. The new generation of biofuel consists not only of ethanol, but also microbial oils. The possibility to produce fatty acids was analyzed using *Fucus vesiculosus* and *Saccharina latissima*. Previous research evaluating the possibility to use brown algae extracts focused on hydrolysates obtained with acidic or enzymatic hydrolysis, therefore obtaining glucose in the final extract [15]. Additionally, a study on *Sargassum* was conducted, to analyze its potential as a source of alginate and sugars for de novo biodiesel production by yeast [17]. Contrary to that examination, only mannitol was extracted from the dry algae biomass in this study, in a process that additionally did not require any pretreatment or adjustments, such as milling, autoclaving prior to extraction, or addition of acid or enzymes. This strategy therefore makes it more cost-efficient in potential larger scale applications. Similarly to the previous study, the amount of lipids was analyzed. The results of this study indicate higher overall biomass levels for all of the modified strains, excluding the *DGA1* overexpressing strain. The percentage of accumulated fatty acids from macroalgae-based medium in the AJD *Δeyd1 Dga1*strain exceeded 30%, compared to 10% previously reported by Dobrowolski et al. [15]. Up-scaling the process allowed for production of lipids at level of 20% of dry yeast biomass. The utilization of carbon source was also observed within a similar timeframe. Despite the modified strain obtaining 30% of biomass in the form of lipids, this value does not approach the overall potential of *Y. lipolytica* to store lipids while growing on different agricultural waste materials, where the percentage can exceed 50% [10]. Those materials, heavily processed to extract carbohydrates, are usually rich in glucose, whereas mostly mannitol was observed in the extracts of *F. vesiculosus* used in this study. The ability to store large quantities of lipids is not yet fully utilized in the case of lipid production on algal extracts, and therefore further modifications are required. Brown seaweeds are known to produce phlorotannins, which are proven to have antiviral, antibacterial, antifungal and larvicidal activities; hence there is a possibility of a negative impact of the brown algae extract on growth of the yeast [30]. This process was not observed in *Y. lipolytica*, highlighting its robustness.

Analysis of lipid production by the oleaginous yeast *Cutaneotrichosporon oleaginosus* on brown algae hydrolysate provided similar yield of overall lipid content (3.8 g/L compared to 3.95 g/L for the AJD *Δeyd1 Dga1* strain in deep-well plates); however, the carbohydrate content of brown algae hydrolysate of *Laminaria digitata* was rich in glucose. Mannitol constituted only 9.2 ± 0.04 g/L, while glucose was reported at 19.0 ± 0.1 g/L, with addition of xylose (4.4 ± 0.02 g/L). Moreover, the brown algae were enzymatically pretreated [42], which is not necessary in the case of utilization of mannitol extracts by *Y. lipolytica*. The level of accumulated fatty acids was therefore similar, while AJD *Δeyd1* and AJD *Δeyd1 Dga1* strains utilized mostly mannitol and not glucose. That results in a higher production yield for the modified *Y. lipolytica* strain used in this study.

The profile of the produced fatty acids by the AJD *Δeyd1 Dga1* strain in algal mannitol medium shows that the majority of the overall lipids come in the form of monounsaturated (MUFA) oleic acid, which is one of the most abundantly produced fatty acids in most conditions and carbon sources [12], and palmitoleic acid, constituting more than 75% of all produced fatty acids. Saturated fatty acids do not exceed 15% of the overall production between both palmitic and stearic acids. Those results are also reflective of attempts to produce cocoa butter substitutes by using *Y. lipolytica*, where both oleic and palmitoleic acid constituted more than 79% of the overall fatty acids, while unsaturated fatty acids (MUFA) and polyunsaturated fatty acids (PUFA) did not exceed 15% [32]. In contrast, the production of fatty acids by *Y. lipolytica* on sugars from *Sargassum* yielded mostly saturated fatty acids, predominantly palmitic and stearic acids (both around 30% of the overall production), while MUFAs were the second largest fraction [17].

A possibility of co-culture of oleaginous yeast and algae for lipid production was also studied [7], although in that case microalgae were utilized. Algae provided O_2_ for the yeast, which can be an alternative for mechanical aeration. In this study macroalgae biomass was selected as a carbon source. The possibility of utilizing the co-culture strategy in medium containing the brown algae extracts is not beyond the realm of possibility, as both the yeast and the algae benefit from those conditions. The effects of this approach on lipid production need to be validated in the case of *Y. lipolytica* and the potential microalgae partner.

The results of this study provide a possible approach for utilization of brown algal biomass by the yeast *Y. lipolytica*, with improved fatty acid production. This strategy is an interesting alternative for application of brown algae carbohydrates. These organisms are abundant in the environment and rich in carbohydrates that can be utilized for production of value-added products, such as single cell oils. This alternative for biofuel synthesis needs to be further evaluated, as the possibility of obtaining extracts from other algal species, further modifications in fatty acid synthesis pathway or co-culture with microalgae can additionally increase the yield of lipid accumulation and prove to be even more profitable. The results of this study provide the potential application of conversion of algal mannitol into fatty acids, as opposed to production of bioethanol, which further widens the scope of brown algae biomass application and its role as a fourth generation of biomass.

## Conclusions

The modified *Y. lipolytica* strain, with knockout of the *EYD1* gene, is a potential chassis for utilization of brown algae extracts, with the ability to convert the carbohydrates from those extracts to fatty acids. The innate ability to take up many carbon sources, coupled with the altered mannitol utilization, provides a good host for microbial cell oil production on a fourth generation of biomass. These characteristics are an important step towards generation of biofuels from algal biomass, which is believed to be an important part of the solution of replacing the diminishing non-renewable fossil fuels. Further modifications in the fatty acid synthesis pathway can lead to increased accumulation of lipids in the cells or an overall higher conversion rate. Coupling the features of the yeast *Y. lipolytica*, such as utilization of many unconventional carbon sources, ability to withstand harsh environmental conditions and lipid production, has proved to be an interesting strategy for single cell oil production on waste biomass. Additionally, the possibility to alter the fatty acid production profile in *Y. lipolytica* is also extremely important, as the application of those single cell oils can vary depending on that factor. The better understanding of mannitol metabolism will also increase the potential for utilization of other microorganisms that are applied in industrial production of value-added products as well as the in-depth analysis of this pathway in general.

## Electronic supplementary material

Below is the link to the electronic supplementary material.

Supplementary Material 1

## Data Availability

No datasets were generated or analysed during the current study.
