# Supplementary material for: Engineered yeast Yarrowia lipolytica as a chassis for biosynthesis of fatty acids from mannitol and macroalgal biomass extracts
Source: Microb Cell Fact. 2025 Mar 26;24:72. doi: 10.1186/s12934-025-02699-9 (PMC11938780; doi:10.1186/s12934-025-02699-9)
Supplement: Supplementary file 1 — Supplementary Material 1 [file 12934_2025_2699_MOESM1_ESM.pdf]

Supplementary Data

Engineered yeast *Yarrowia lipolytica* as a chassis for biosynthesis of fatty acids from mannitol and macroalgal biomass extracts

Mateusz Szczepańczyk[1], Dorota A. Rzechonek[1,2], Adam Dobrowolski[1], Aleksandra M. Mirończuk[1]*

[1] Wrocław University of Environmental and Life Sciences, Institute of Environmental Biology, Laboratory for Biosustainability, 5b Kozuchowska St., 51-631, Wroclaw, Poland

[2]Department of Life Sciences (LIFE), Chalmers University of Technology, Kemivägen 10. SE-412 96 Göteborg, Sweden

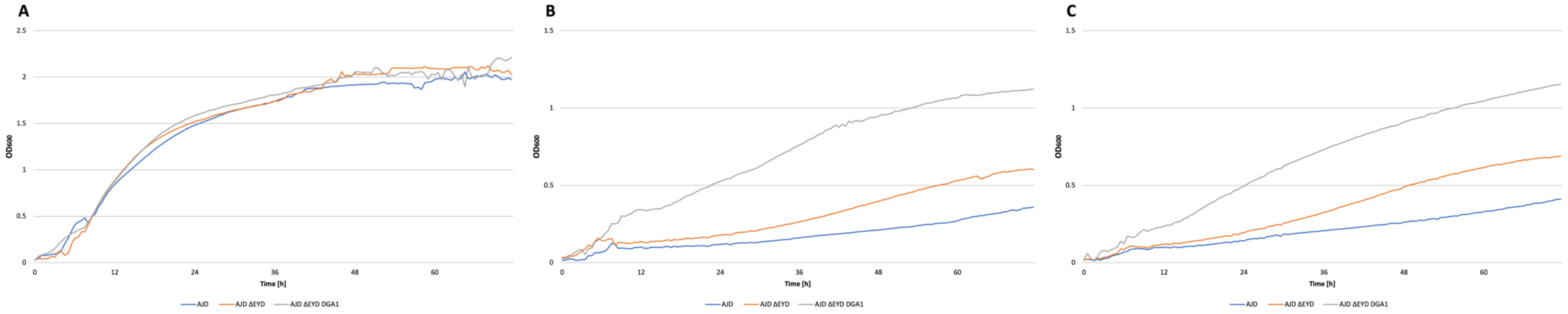

Figure 1. Growth curves for parental strain AJD (blue), strain with the knockout of *EYD1* gene (orange) and the knockout strain with additional overexpression of *DGA1* gene (gray) in rich YPD medium (A) on minimal medium with 2% mannitol (B) and minimal medium supplemented with 10% mannitol (C).
